# Implications of CRNDE in prognosis, tumor immunity, and therapeutic sensitivity in low grade glioma patients

**DOI:** 10.1186/s12935-023-02930-w

**Published:** 2023-05-16

**Authors:** Chen Yang, Yingchuan Jiang, Fan Hu, Qiuping Li, Biao Qi

**Affiliations:** 1 Department of Neurosurgery, Zhongshan Hospital, Fudan University (Xiamen Branch), Xiamen, 361015 Fujian China; 2 Department of Neurosurgery, Zhongshan Hospital, Fudan University, Shanghai, 200032 China

**Keywords:** CRNDE, Low grade glioma, Prognosis, Tumor immunity, Therapeutic sensitivity

## Abstract

**Background:**

Colorectal tumor differentially expressed (CRNDE) is specifically expressed in human brains and is the most highly expressed lncRNA in gliomas. Nevertheless, its implications in low grade glioma (LGG) are still indistinct. This study presented systematic analyses of CRNDE in LGG biology.

**Methods:**

We retrospectively retrieved TCGA, CGGC and GSE16011 LGG cohorts. Survival analysis was conducted for evaluating the prognostic significance of CRNDE in LGG. A CRNDE-based nomogram was established, and its predictive performance was verified. Signaling pathways underlying CRNDE were analyzed through ssGSEA and GSEA approaches. The abundance of immune cells and activity of cancer-immunity cycle were estimated with ssGSEA approach. Immune checkpoints, HLAs, chemokines, and immunotherapeutic response indicators (TIDE, and TMB) was quantified. U251 and SW1088 cells were transfected with specific shRNAs of CRNDE, and flow cytometry (apoptosis) and western blot (β-catenin and Wnt5a) assays were conducted.

**Results:**

Up-regulated CRNDE was found in LGG, and was linked to unfavorable clinical outcomes. The CRNDE-based nomogram enabled to accurately predict patients’ prognosis. High CRNDE expression was linked to more genomic variations, activity of tumorigenic pathways, tumor immunity (increase in infiltration of immune cells, expression of immune checkpoints, HLAs and chemokines, and cancer-immunity cycle), and therapeutic sensitivity. CRNDE knockdown mitigated malignant phenotypes of LGG cells.

**Conclusions:**

Our study determined CRNDE as a novel predictor for patient prognosis, tumor immunity and therapeutic response in LGG. Assessment of CRNDE expression is a promising approach for predicting the therapeutic benefits of LGG patients.

**Supplementary Information:**

The online version contains supplementary material available at 10.1186/s12935-023-02930-w.

## Background

Gliomas are the dominating primary central nervous system malignancies of human brains [1], which are classified as low grade glioma (LGG, composed of astrocytoma, oligoastrocytoma, and oligodendroglioma; grade I, II, and III) and glioblastoma (grade IV) [2]. LGG is a relatively slow-growing, invasive, progressive brain tumor, which usually occurs in the third and fourth decade of life [3]. Surgical resection, radiotherapy, and temozolomide chemotherapy all exert roles in the clinical management of LGG [3]. Nevertheless, the sequence and optimal timing are still debated. Advance in the tumor microenvironment and the brain’s immune response have inspired immunotherapy research. However, the validation of classic PD-1/PD-L1 inhibitors failed in phase III clinical trials among glioma patients [4]. More effective molecular targets are required against LGG.

Colorectal tumor differentially expressed (CRNDE), long noncoding RNA (lncRNA), is expressed in specific regions of the human brain as well as is the most highly expressed lncRNA in gliomas [5]. Experimental evidence has demonstrated the roles of CRNDE in gliomas. For instance, CRNDE triggers growth and invasion of glioma cells via mTOR signaling [5]. Knockdown of CRNDE heightens temozolomide sensitivity via autophagy in glioblastoma [6]. CRNDE facilitates malignant progression of gliomas through inactivating miR-384/PIWIL4/STAT3 signaling [7]. Up-regulated CRNDE acts as an unfavorable prognostic indicator of glioblastoma patients [8]. Nevertheless, the functions and possible mechanisms of CRNDE in LGG have not been expounded. Our study was conducted for uncovering the implications of CRNDE in prognosis, tumor immunity as well as therapeutic sensitivity in LGG patients. In addition, in vitro experiments were conducted to verify the roles of CRNDE in LGG progression.

## Materials and methods

### Data retrieval

RNA-seq profiling (fragments per kilobase per million (FPKM)) and matched clinical information of pan-cancer tissue samples were obtained from The Cancer Genome Atlas (TCGA) database utilizing TCGAbiolinks package [9]. The FPKM value was converted into transcripts per kilobase million (TPM) value. Single nucleotide variants (SNVs) (mutation annotation format), copy number variations (CNVs), aneuploidy score, tumor mutational burden (TMB), cancer-testis antigen (CTA) score, homologous recombination defects, intratumor heterogeneity, SNV neoantigens, SCNA level, immune subtypes and stemness score (mRNA expression based-index (mRNAsi)) of LGG samples were also retrieved from TCGA database. Transcriptome data and matched clinical data of LGG patients were also obtained from Chinese Glioma Genome Atlas (CGGC) and GSE16011, as an external validation. Additional file 1: Fig. S1 illustrates the schematic diagram of our study design.

### Genomic variation analysis

The mutational landscape was visualized through maftools package [10]. CNVs were analyzed utilizing GISTIC 2.0 for identifying arm- or focal-level variations in TCGA LGG samples [11].

### Functional enrichment analyses

The ssGSEA approach was applied for estimating the standardized enrichment score utilizing GSVA package [12]. The gene expression values of LGG samples were ranked and the rest of the genes was used for generating enrichment scores in a specific signature. The gene set files “c5.go.bp.v7.5.1.symbols”, “c2.cp.kegg.v7.5.1.symbols” and “” and hallmark were obtained from the Molecular Signatures Database (MSigDB) [13]. The markers in known biological pathways were collected from previously published literature [14]. Gene set enrichment analysis (GSEA) of TCGA LGG samples was carried out utilizing GSEA software [15].

### Analysis of tumor immunity signatures

Tumor immunity signatures were assessed in two aspects. (1) The expression of immune checkpoints, human leukocyte antigen (HLA) genes and chemokines was measured. (2) ssGSEA was utilized for quantifying the abundance of immune cell fractions on the basis of the gene sets from Charoentong’s study [16]. The fractions of stromal and immune cell types in LGG tissues were computed with the Estimation of STromal and Immune cells in MAlignant Tumor tissues using Expression data (ESTIMATE) algorithm [17].

### Evaluation of therapeutic response

The gene sets of seven steps within the cancer-immunity cycle were collected from previously published literature [18]. Above events were scored by ssGSEA utilizing gene expression for each LGG tissue. Tumor Immune Dysfunction and Exclusion (TIDE) tool (http://tide.dfci.harvard.edu/) was utilized for calculating the TIDE score [19]. High TIDE score predicts poor benefit from immunotherapy. TMB each megabase was computed through the ratio of the total number of mutations in each LGG tissue to the genome size of the coding region (40 Mb), as a biomarker of immunotherapeutic response. Drug response data were collected from the GDSC dataset [20]. The 50% inhibiting concentration (IC50) values of therapeutic compounds were inferred utilizing pRRophetic algorithm [21].

### Cell culture and transfection

U251 and SW1088 cells (ATCC) were grown in DMEM supplemented with 10% fetal bovine serum (Sigma Aldrich) in a humidified atmosphere of 5% CO_2_ at 37 °C. Short hairpin RNA (shRNA) targeting CRNDE or scramble shRNA (Genechem) was cloned into lentiviral vectors, followed by transfection into U251 and SW1088 cells for 3 days and exposure to 4 μg/mL puromycin for one week.

### RT-qPCR

Total RNA was extracted from cells utilizing TRIzol reagent (Beyotime), which was quantified via Nanodrop 1000. cDNA was reverse-transcribed from 500 ng extracted RNA utilizing PrimerScript RT Master Mix (Takara) and diluted at 1:20 with DEPC water. RT-qPCR was then carried out through SYBR Green II Mixture (TaKaRa) in an ABI 7900 system (ABI). 2^−ΔΔCt^ method was utilized to calculate the relative expression of CRNDE with GAPDH as an internal reference. The primer sequences were as follows: CRNDE, 5'-TGAAGGAAGGAAGTGGTGCA-3' (forward), 5'-TCCAGTGGCATCCTACAAGA-3' (reverse); GAPDH, 5'-GGTCTCCTCTGACTTCAACA-3' (forward), 5'-GTGAGGGTCTCTCTCTTCCT-3' (reverse).

### Flow cytometry

Cell apoptosis detection kit (KTA0002; Abbkine) was adopted for flow cytometry. Briefly, cell suspension was incubated with 5 μL Annexin V-AbFluor™ 488 binding and 2 μL PI at room temperature and away from light for 15 min. After adding 400 μL 1× Annexin V buffer, apoptotic level was measured utilizing Flow cytometer (Beckman).

### Western blot

Cells were lysed in RIPA buffer (Beyotime), followed by protein concentration measurement utilizing BCA method. Equal protein amount was loaded onto 10% SDS-PAGE and transferred onto PVDF membranes. After blockade in 5% non-fat milk for one hour, the membranes were incubated with primary antibody of β-catenin (1:1000; ABM0057; Abbkine), Wnt5a (1:1000; 55184-1-AP; Proteintech), or GAPDH (1:3000; ab8245; Abcam) at 4 °C overnight, followed by secondary antibody incubation. The membranes were developed, and visualized via gel imaging system (Bio-rad).

### Statistical analysis

Univariate Cox regression models were established for computing hazard ratio (HR) and confidence interval (CI) of CRNDE expression. CRNDE expression and clinical variables were employed for univariable and multivariate models. The forest plots were drawn to visualize the above results. A nomogram was established based on independent prognostic factors. Concordance index (C-index), calibration curves, receiver operating characteristic (ROC) curves and decision curve analyses (DCA) were utilized for evaluating the predictive power of the nomogram. Statistical significance in overall survival (OS), disease-free survival (DFS), progression-free survival (PFS), and disease-specific survival (DSS) analysis was estimated utilizing log-rank test. The predictive capacity of CRNDE expression for survival was demonstrated via ROC curves, followed by calculation of area under the curve (AUC) value. Two groups with non-normally distributed data were compared with Wilcoxon test, with Student’s t-test for normally distributed data. Correlation coefficients were evaluated with Spearman correlation test. All statistical analyses were implemented utilizing R (version 4.1.1). Significant P-value was noted including: ns > 0.05, * < 0.05, ** < 0.01, *** < 0.001 and **** < 0.0001.

## Results

### Expression and prognostic significance of CRNDE in LGG

**Fig. 1:**
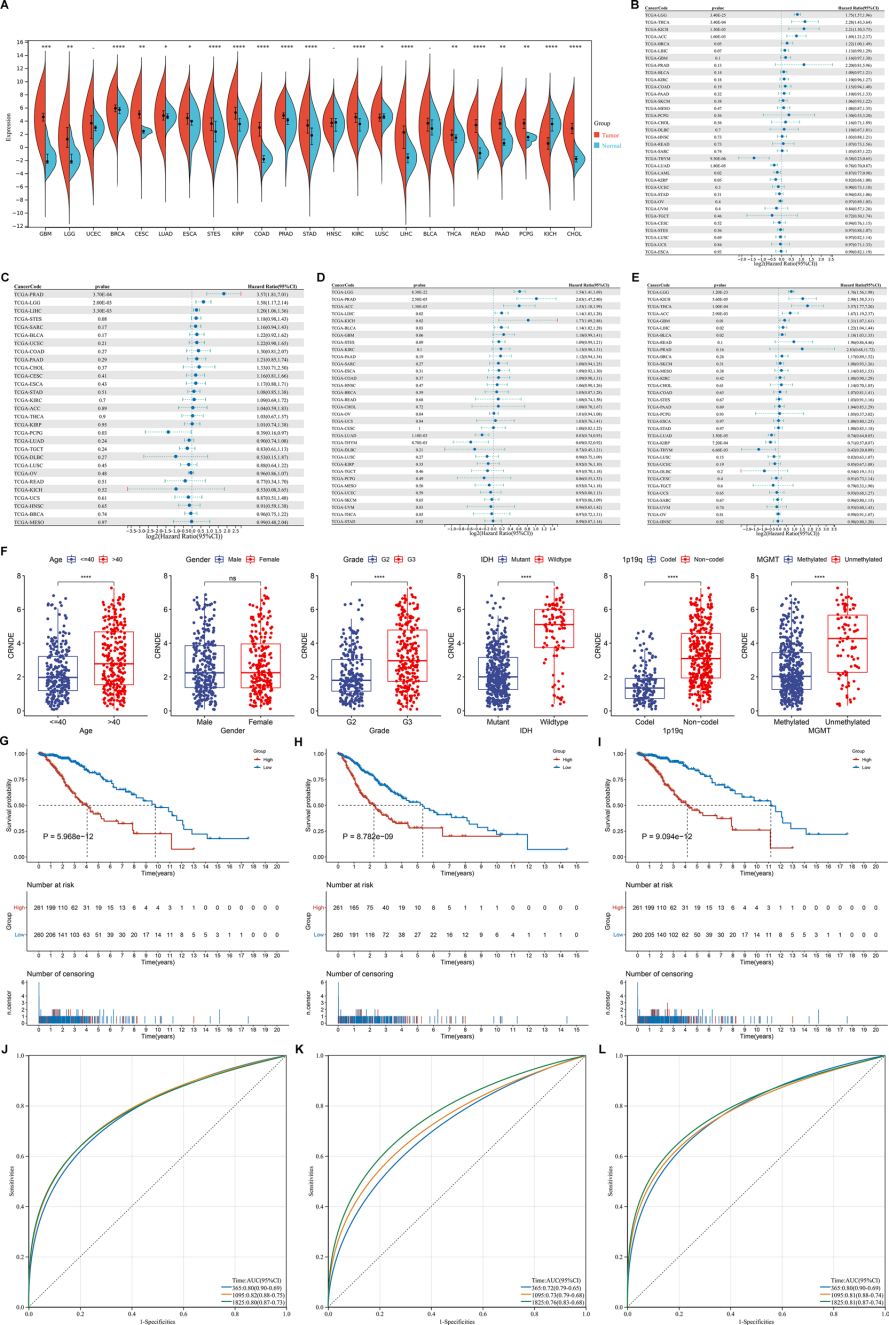
Expression and prognostic significance of CRNDE in LGG. **A** CRNDE expression in pan-cancer primary tumors (orange) and adjacent normal tissues (blue) in TCGA datasets. **B**–**E** Forest diagrams for the univariate Cox regression analyses on CRNDE expression with **B** OS, **C** DFS, **D** PFS, and **E** DSS of TCGA LGG patients. **F** Differences in CRNDE expression between different clinical variables, including age ≤ 40 versus > 40; male versus female; grade G2 versus G3; mutant versus wild type IDH; codel versus non-codel 1p19q; methylated versus unmethylated MGMT. **G**–**I** Kaplan–Meier curves of **G** OS, **H** PFS and **I** DSS for TCGA LGG patients with high or low CRNDE expression. **J**–**L** ROC curves at 1-, 3- and 5-year **J** OS, **K** PFS and **L** DSS for CRNDE expression in TCGA LGG cohort

We firstly assessed CRNDE expression in TCGA pan-cancer tissue samples. Most primary tumors (including glioblastoma (GBM), LGG, breast cancer (BRCA), cervical cancer (CESC), lung adenocarcinoma (LUAD), esophageal cancer (ESCA), stomach and esophageal carcinoma (STES), kidney renal papillary cell carcinoma (KIRP), colon adenocarcinoma (COAD), prostate adenocarcinoma (PRAD), stomach adenocarcinoma (STAD), kidney renal clear cell carcinoma (KIRC), lung squamous cell carcinoma (LUSC), liver hepatocellular carcinoma (LIHC), thyroid carcinoma (THCA), rectum adenocarcinoma (READ), pancreatic adenocarcinoma (PAAD), pheochromocytoma and paraganglioma (PCPG), CHOL) had higher CRNDE expression in comparison to adjacent normal tissues (Fig. 1A). Through univariate Cox regression analyses, prognostic value of CRNDE was evaluated across pan-cancer. It was found that CRNDE expression acted as a risk factor of OS (Fig. 1B), DFS (Fig. 1C), PFS (Fig. 1D), and DSS (Fig. 1E) of LGG patients, indicating a close relationship between CRNDE and LGG prognosis. Thus, we further focused on the role of CRNDE in LGG. Associations between CRNDE expression and clinical variables were evaluated both in TCGA and CGGC datasets. In TCGA dataset, higher CRNDE expression was found in > 40 versus ≤ 40, grade G3 versus G2, wild-type versus mutant IDH, non-codel versus codel 1p19q, and unmethylated versus methylated MGMT, but without significant difference between male and female patients (Fig. 1F). In CGGC dataset, higher CRNDE expression was found in grade G3 versus G2, wild-type versus mutant IDH, and non-codel versus codel 1p19q, but without significant difference in > 40 versus ≤ 40, male versus female, or unmethylated versus methylated MGMT (Additional file 2: Fig. S2A). Additionally, high CRNDE expression group presented worse OS (Fig. 1G), PFS (Fig. 1H) and DSS (Fig. 1I) outcomes than low CRNDE expression group in TCGA dataset. ROC curves demonstrated that CRNDE expression enabled to accurately predict LGG patients’ 1-, 3- and 5-year OS (Fig. 1J), PFS (Fig. 1K) and DSS (Fig. 1L). Consistently, poorer OS outcome was found in patients with high CRNDE expression (Additional file 2: Fig. S2B), and CRNDE expression could predict patients’ survival (Additional file 2: Fig. S2C) in CGGC cohort. The similar survival difference between high and low CRNDE expression groups (Additional file 2: Fig. S2D) and the prediction performance of CRNDE expression (Additional file 2: Fig. S2E) were proven in GSE16011 cohort.

### Establishment of a CRNDE-based nomogram for LGG

**Fig. 2:**
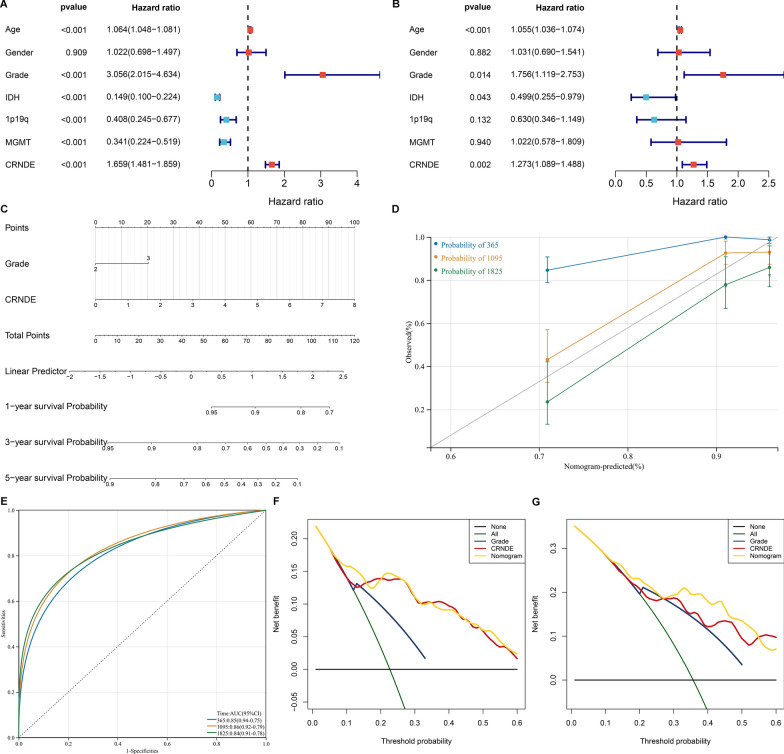
Establishment of the CRNDE-based nomogram for LGG in TCGA cohort. **A**, **B** Forest diagrams of uni- and multivariate Cox regression analyses of CRNDE and clinical variables with prognosis. **C** Nomogram of CRNDE and grade. The total points projected on the bottom scales indicate the 1-, 3 and 5-year survival probabilities. **D** Calibration curves for predicting 1-, 3- and 5-year survival probabilities. **E** ROC curves at 1-, 3- and 5-year survival. **F**, **G** DCA curves for intuitively evaluating the clinical benefits and application of the nomogram. The abscissa represents the 3- and 5-year survival threshold probabilities, and the ordinate represents the estimated net benefits

According to univariate Cox regression analyses, in TCGA dataset, age, grade, and CRNDE were risk factors of LGG prognosis, while IDH, 1p19q, and MGMT were protective factors (Fig. 2A). Subsequent multivariate Cox regression analyses showed that age, grade, and CRNDE were independent risk factors of LGG prognosis (Fig. 2B). To facilitate the clinical application, the nomogram was then established for predicting 1-, 3-, and 5-year survival through totaling the points determined on the points scale for CRNDE and clinical parameters (Fig. 2C). Calibration curves showed that the nomogram-predicted survival probabilities were highly consistent with actual survival (Fig. 2D). The AUC values at 1-, 3-, and 5-year survival exceeded 0.80, demonstrating the high accuracy of the nomogram (Fig. 2E). DCA results confirmed that LGG patients could clinically benefit from the nomogram at 3- (Fig. 2F) and 5-year (Fig. 2G) survival threshold probabilities.

Consistently, in GSE16011 dataset, CRNDE was proven as an independent risk factor of LGG prognosis (Additional file 3: Fig. S3A, B). The CRNDE-based nomogram was also established (Additional file 3: Fig. S3C), which could accurately predict patient survival (Additional file 3: Fig. S3D), and had clinical benefits at 1- (Additional file 3: Fig. S3E), 3- (Additional file 3: Fig. S3F) and 5-year (Additional file 3: Fig. S3G) survival threshold probabilities. The independency of CRNDE in prognosis prediction was also demonstrated in CGGC cohort (Additional file 4: Fig. S4A, B). As expected, the CRNDE-based nomogram enabled to accurately predict patient survival according to calibration curves (Additional file 4: Fig. S4C), and ROC curves (Additional file 4: Fig. S4D). DCA results at 3- (Additional file 4: Fig. S4E) and 5-year (Additional file 4: Fig. S4F) survival threshold probabilities also proven the excellent clinical benefits. The C-index of the model was > 0.7 in TCGA, GSE16011 and CGGC cohorts. Altogether, the CRNDE-based nomogram might be a reliable tool for prognosis prediction of LGG.

### Associations between CRNDE and genomic variations in LGG

**Fig. 3:**
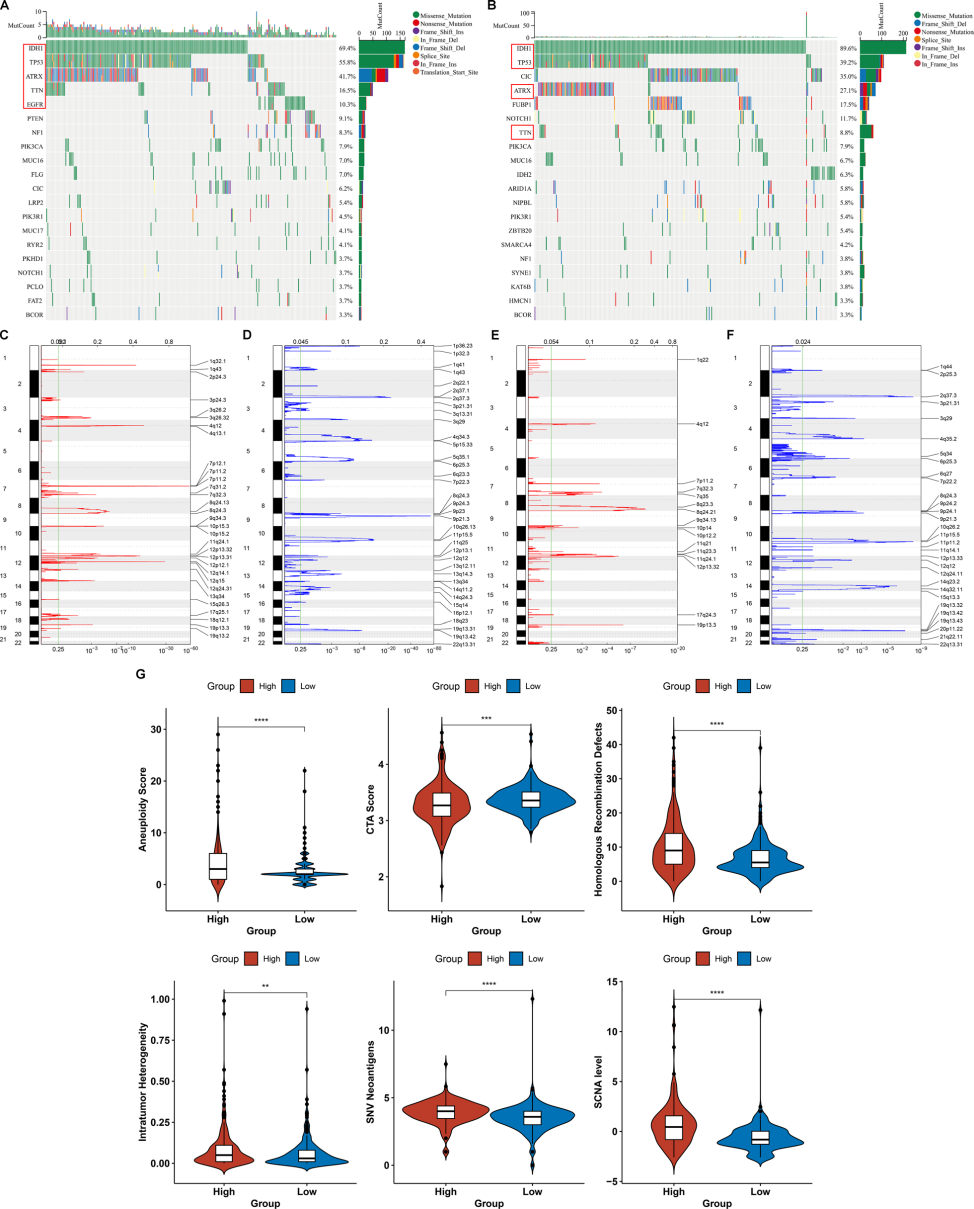
Associations between CRNDE and genomic alterations in LGG. **A**, **B** Waterfall plots for the first mutational events for each TCGA LGG case with **A** high or **B** low CRNDE expression. Statistical diagrams of mutational events of individual genes are displayed in the left panel. Mutation types are marked by unique colors in the right panel. Bar diagram in the top panel represents the number of mutations across cases. **C**, **D** Significantly **C** amplified and **D** deleted gene fragments in the high CRNDE expression group. **E**, **F** Significantly **E** amplified and **F** deleted gene fragments in the low CRNDE expression group. The abscissa denotes the CNV fragments on chromosomes, and the ordinate denotes the chromosome number. Red and blue separately represent the amount of CNV gains and losses. The CNV locations on the chromosomes are noted on the right panel. **G** Differences in aneuploidy score, CTA score, homologous recombination defects, intratumor heterogeneity, SNV neoantigens, and SCNA level between high and low CRNDE expression groups

More genomic instability represents a commonly detected hallmark of cancer. Overall, high CRNDE expression group (Fig. 3A) occurred more somatic alterations in comparison to low CRNDE expression group (Fig. 3B). For identifying the mutational difference between two groups, the first 20 mutated genes were analyzed. Mutated IDH1 was more frequent for samples with low CRNDE expression, while mutated TP53, ATRX, TTN, EGFR, etc. were more frequent in high CRNDE expression group (Fig. 3A, B). The difference in arm-level CNV gains or losses was evaluated. Overall, high CRNDE expression samples had more copy number gains (Fig. 3C) and losses (Fig. 3D) in comparison to low CRNDE expression samples (Fig. 3E, F), indicating that CRNDE was positively linked with copy number variations in LGG.

Additionally, this study validated the interactions utilizing aneuploidy score that is an experimental approach for measuring the entire amount of changed chromosome arms [22]. Aneuploidy score was computed as the sum of altered arms on a scale of 0–39 (long and short arms for each non-acrocentric chromosome, and only long arms for chromosomes 13, 14, 15, 21 and 22). Higher aneuploidy score was present in high CRNDE expression group (Fig. 3G). We then assessed the associations between CRNDE expression and tumor immunogenicity, containing CTA score, homologous recombination defects, intratumor heterogeneity, SNV neoantigens and SCNA level, thereby characterizing the multi-dimensional maps of the immuno-oncology landscape. High CRNDE expression group displayed relatively higher homologous recombination defects, intratumor heterogeneity, SNV neoantigens, and SCNA level, and lower CTA score in comparison to low CRNDE expression group (Fig. 3G).

### Signaling pathways underlying CRNDE in LGG

**Fig. 4:**
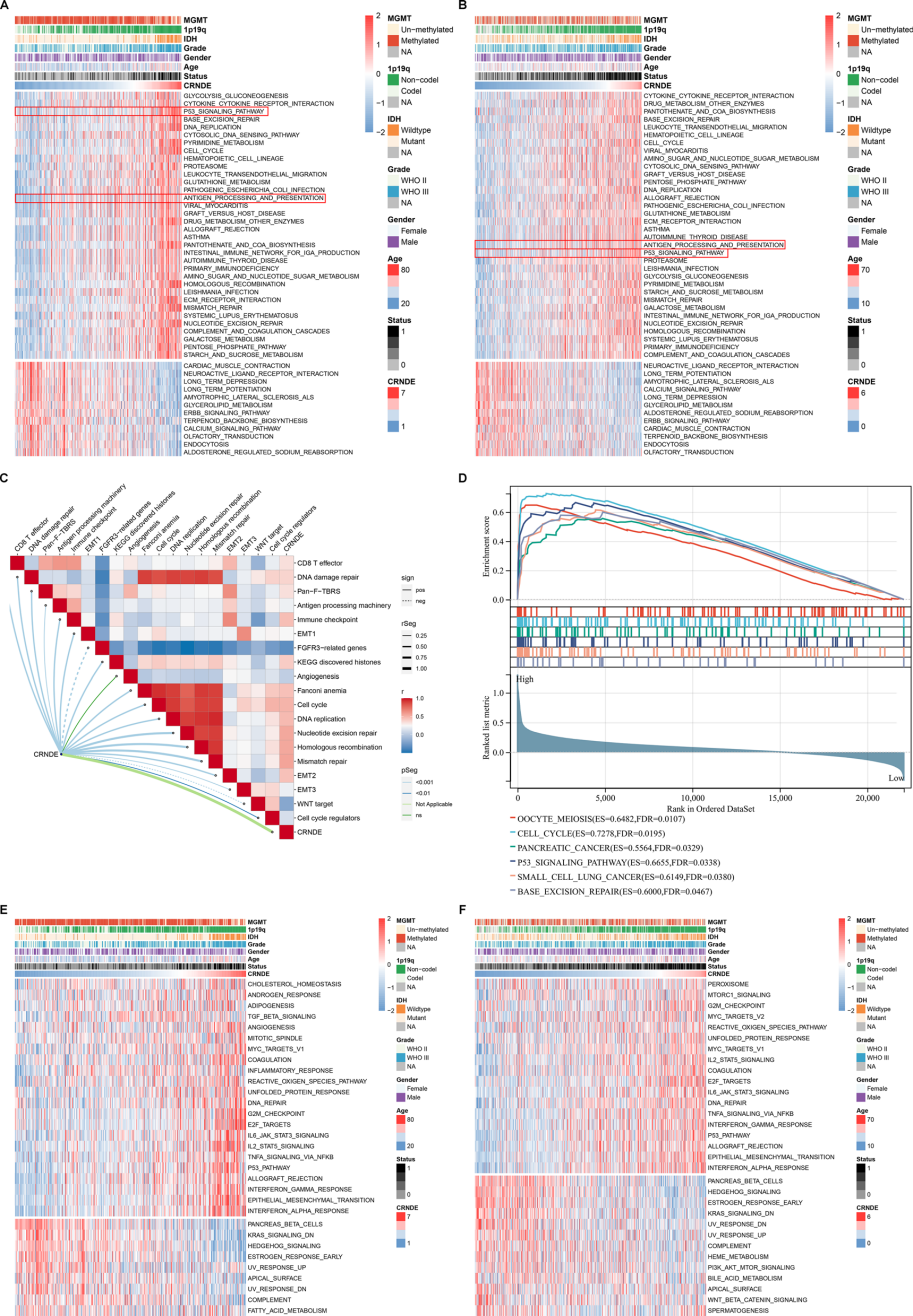
Signaling pathways underlying CRNDE in LGG. **A**, **B** The activity of GO and KEGG pathways in **A** TCGA and **B** CGGC LGG tissues with high or low CRNDE expression. **C** Associations between CRNDE expression and known biological processes. **D** GSEA for the pathways activated in high CRNDE expression group. **E**, **F** The activity of hallmark pathways in **E** TCGA and **F** CGGC LGG tissues with high or low CRNDE expression

The molecular mechanisms underlying CRNDE were then assessed. Tumorigenic pathways (p53 pathway, etc.) and tumor immunity (antigen processing and presentation, etc.) displayed the higher activity in high CRNDE expression group versus low CRNDE expression group in TCGA dataset (Fig. 4A), which were proven in CGGC datasets (Fig. 4B). Additionally, CRNDE expression was positively correlated to tumor immunity, stromal activation (pan-F-TBRS, EMT1-3, angiogenesis, etc.), cell cycle progression, and DNA damage repair in TCGA LGG samples (Fig. 4C). GSEA indicated that cell cycle, and tumorigenic pathways (p53 pathway, pancreatic cancer, and small cell lung cancer) were activated in high CRNDE expression group compared with low CRNDE expression group (Fig. 4D). It was also confirmed the close relationships of CRNDE with most hallmark pathways both in TCGA (Fig. 4E) and CGGC cohorts (Fig. 4F).

### Associations between CRNDE and tumor immunity of LGG

**Fig. 5:**
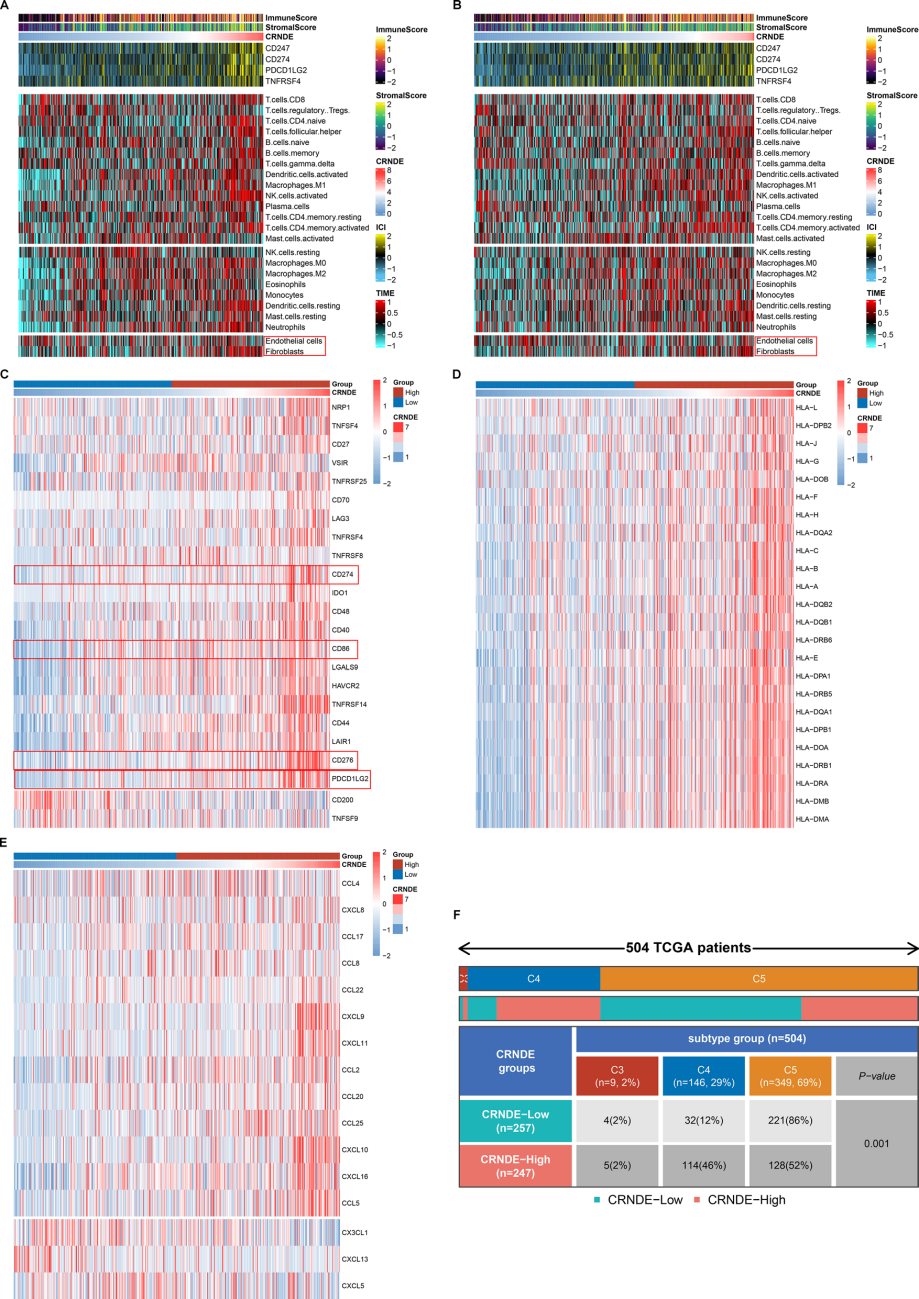
Associations between CRNDE and tumor immunity in LGG. **A**, **B** Heatmaps for the infiltration of immune and stromal cells, stromal and immune scores, and the expression of immune checkpoints in **A** TCGA and **B** CGGC LGG samples. **C**–**E** The expression of **C** immune checkpoints, **D** HLAs, and **E** chemokines across TCGA LGG samples with high or low CRNDE expression. **F** Distribution of immune subtypes across the two groups from TCGA dataset

Tumor immunity acts as a key dominator in tumor growth as well as patients’ survival. Hence, we investigated the impact of CRNDE on tumor immunity of LGG. Using ESTIMATE approach, immune score (indicating the proportions of immune cell populations), and stromal score (indicating the proportions of stromal cell populations) were computed across TCGA (Fig. 5A) and CGGA (Fig. 5B) LGG samples, respectively. Both in the two cohorts, high CRNDE expression was positively correlated to increased immune and stromal scores (Fig. 5A, B). Through implementing ssGSEA, 22 immune cell compositions were deconvoluted. The abundance of most tumor-infiltrating immune cell populations was positively linked to CRNDE expression (Fig. 5A, B). Moreover, we found the positive interactions between CRNDE expression and the abundance of endothelial cells and fibroblasts (Fig. 5A, B). Furthermore, this study evaluated immune molecular features associated with CRNDE expression. High CRNDE expression group displayed the increased expression of immune checkpoints such as CD274 (PD-L1), PDCD1LG2 (PD-L2), CD86, and CD276 (Fig. 5C). Moreover, positive correlations of CRNDE expression with HLA molecules (Fig. 5D) and chemokines (Fig. 5E) were found across LGG samples. Patients in high CRNDE expression group had more lymphocyte depleted phenotype (C4) and more immunologically quiet phenotype (C5) in comparison to those in low CRNDE expression group [23] (Fig. 5F). Altogether, above analyses demonstrated the role of CRNDE in tumor immunity of LGG.

### Evaluation of the predictive value of CRNDE for immunotherapy response of LGG

**Fig. 6:**
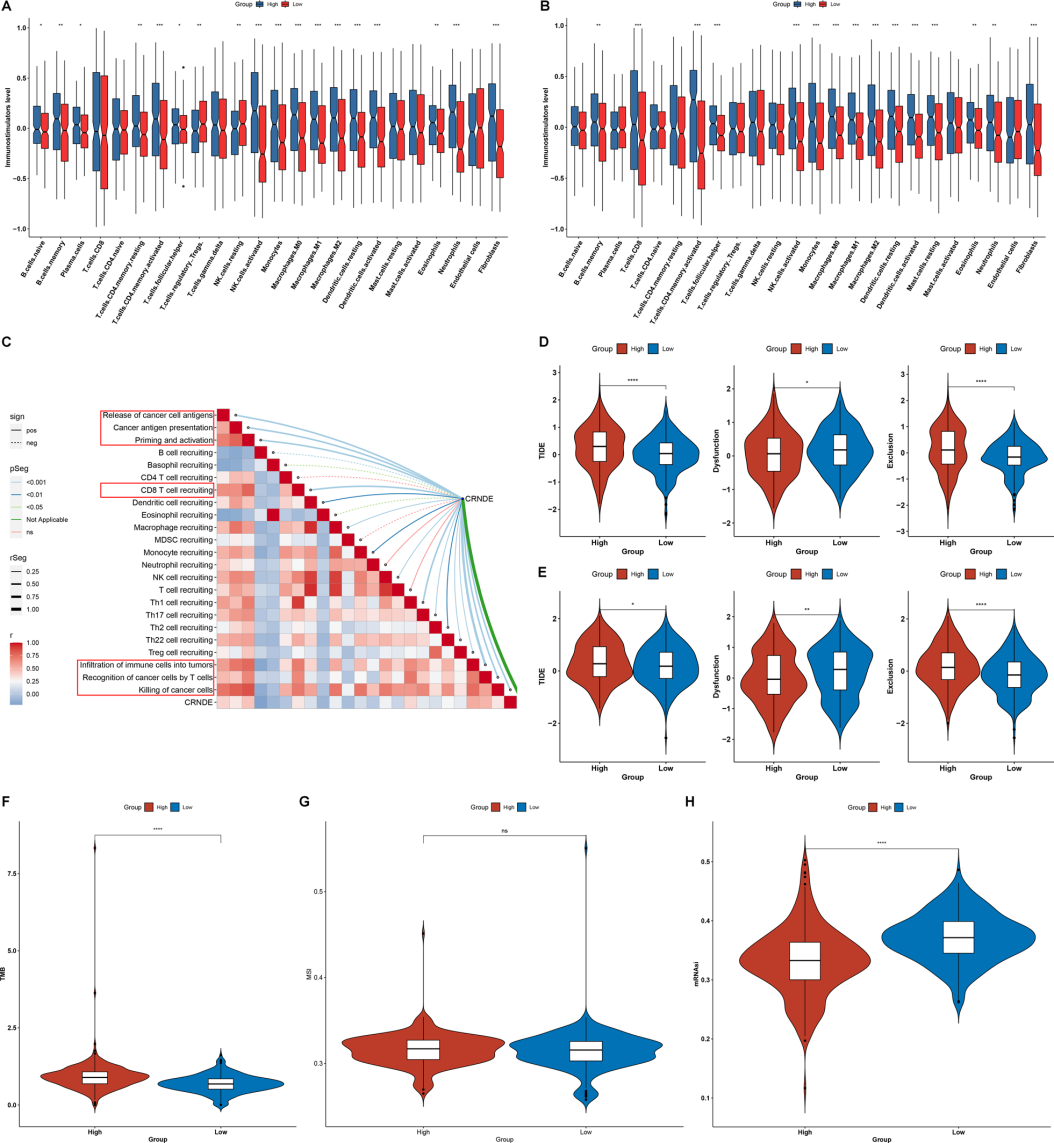
Evaluating the predictive value of CRNDE for immunotherapy response of LGG. **A**, **B** The abundance of immune cell populations of **A** TCGA and **B** CGGC LGG samples with high or low CRNDE expression. **C** Associations between CRNDE expression and seven steps within the cancer-immunity cycle across TCGA LGG samples. **D**, **E** TIDE, dysfunction and exclusion scores for **D** TCGA and **E** CGGC LGG samples with high or low CRNDE expression. **F** TMB, **G** MSI, and **H** mRNAsi in the two groups from TCGA dataset

Both in TCGA (Fig. 6A) and CGGC (Fig. 6B) cohorts, high CRNDE expression group displayed the enhanced abundance of most immune cell types (especially monocytes (M0, M1, and M2 macrophages), dendritic cells (resting and activated dendritic cells), NK cells, and T cells) in comparison to low CRNDE expression group. The cancer-immunity cycle comprises seven steps, which can reflect anti-tumor immunity. Through ssGSEA function, we quantified the activity of each step. In Fig. 6C, CRNDE expression was positively correlated to most steps, such as release of cancer cell antigens, cancer antigen presentation, priming and activation, CD8 T cell recruiting, infiltration of immune cells into tumors, recognition of cancer cells by T cells, and killing of cancer cells.

To predict immunotherapeutic response, we computed TIDE of LGG patients on the basis of two main mechanisms of tumor immune escape: dysfunction of T cells in tumor tissues with highly infiltrated cytotoxic T lymphocytes (CTLs) as well as exclusion of T cell infiltrations in tumor tissues with lowly infiltrated CTLs. Both in TCGA and CGGC datasets, high CRNDE expression group had the increased TIDE and exclusion scores and reduced dysfunction score (Fig. 6D, E). Additionally, high CRNDE expression was linked to increased TMB (Fig. 6F). Microsatellite instability (MSI) represents a molecular feature of hypermutated tumors because of defects in mismatch repair genes [24]. However, no difference was found between high and low CRNDE expression groups (Fig. 6G). Evidence demonstrates that mRNAsi correlates to immunotherapeutic response of glioblastoma [25]. Lower mRNAsi was found in high CRNDE expression group (Fig. 6H).

### Prediction of therapeutic sensitivity in high and low CRNDE for LGG

**Fig. 7:**
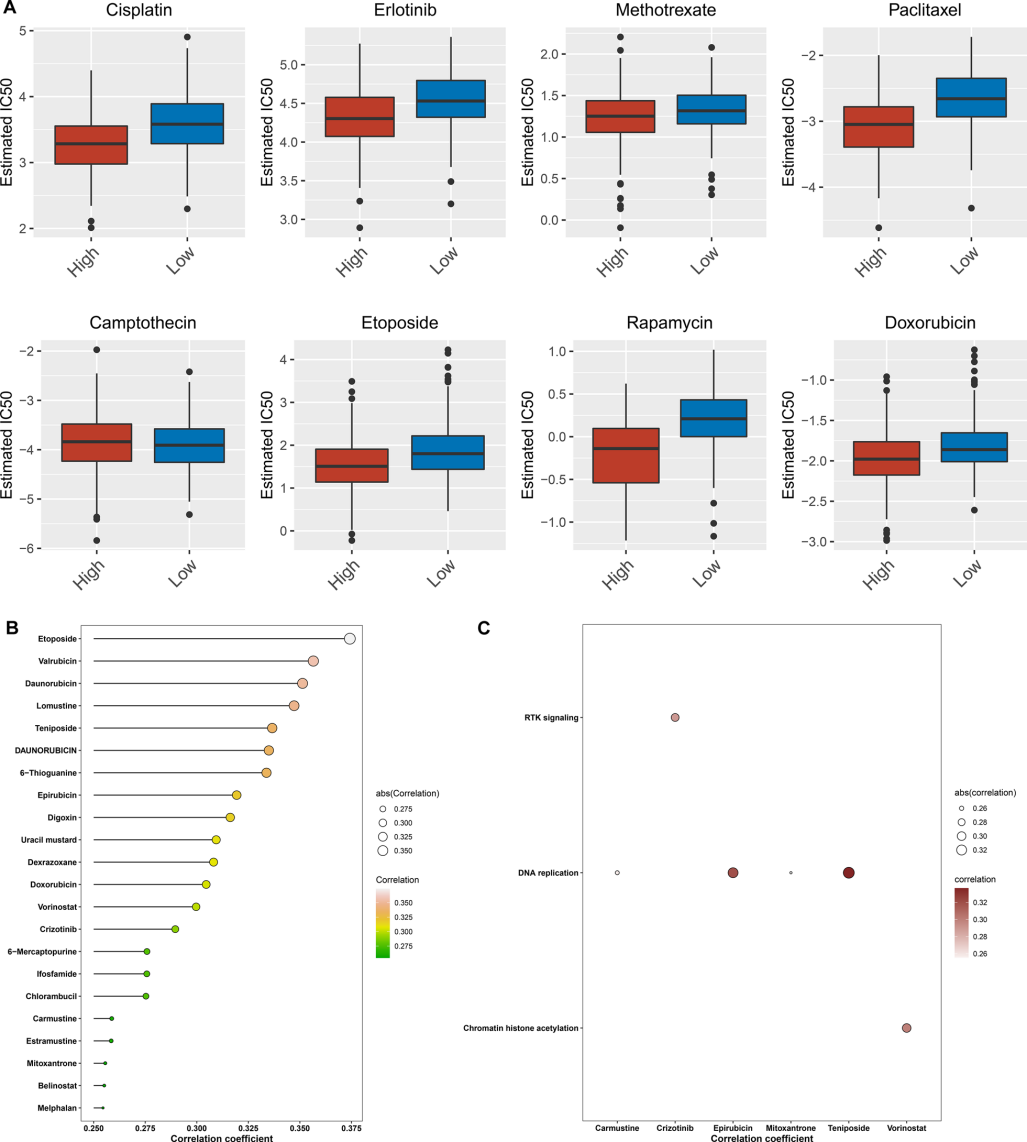
Prediction of therapeutic sensitivity in high and low CRNDE for TCGA LGG patients. **A** Differences in estimated IC50 values of common drugs for high or low CRNDE expression specimens. **B** Interactions of CRNDE expression with GDSC-derived drug sensitivity via Spearman correlation analyses. The length of the horizontal line represents the correlation coefficient. **C** Associations of drugs with KEGG pathways utilizing Spearman correlation analyses

Associations between CRNDE expression and drug sensitivity were further evaluated across TCGA LGG samples. High CRNDE expression group had the higher sensitivity to Cisplatin, Erlotinib, Methotrexate, Paclitaxel, Camptothecin, Etoposide, Rapamycin, and Doxorubicin than low CRNDE expression group (Fig. 7A). This study further assessed the GDSC drug response dataset to determine potential small molecular compounds associated with CRNDE. Drug response of patients with high and low CRNDE expression was estimated according to the AUC values of compounds. Three compounds (Etoposide, Valrubicin, and Daunorubicin) with Spearman’s r > 0.35 were determined via Spearman correlation analyses between CRNDE expression and AUC value (Fig. 7B), indicating that CRNDE expression was correlated to the enhanced sensitivity of above compounds. In addition, the identified drugs were significantly correlated to tumorigenic pathways including DNA replication, RTK signaling and chromatin histone acetylation (Fig. 7C).

### CRNDE contributes to malignant phenotypes of LGG cells

**Fig. 8:**
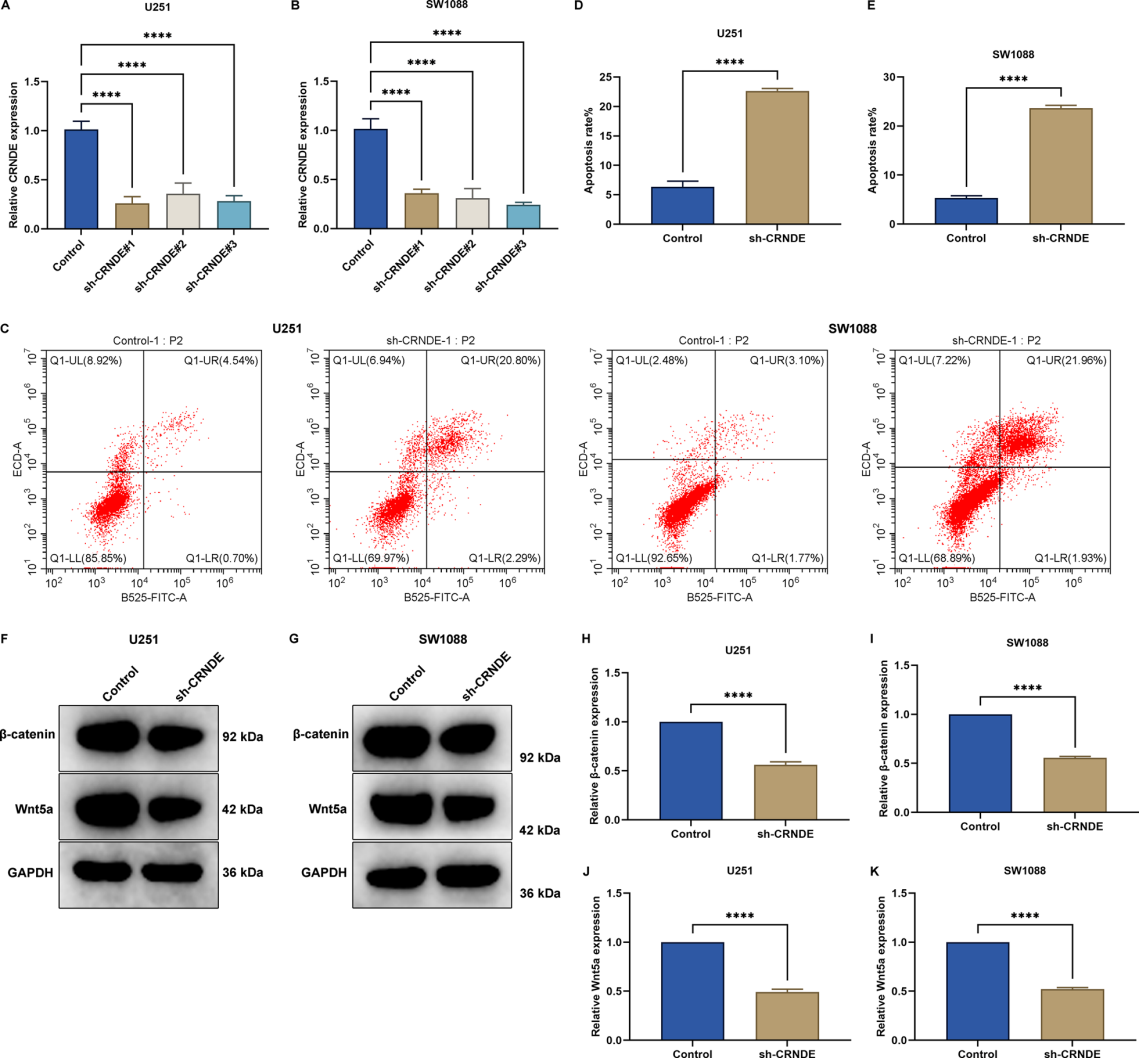
CRNDE contributes to malignant phenotypes of LGG cells. **A**, **B** CRNDE transcript level in U251 and SW1088 cells with CRNDE knockdown. **C**–**E** Apoptotic level of CRNDE-knockout U251 and SW1088 cells. **F**–**K** Protein level of β-catenin and Wnt5a in CRNDE-knockout LGG cells. ****p < 0.0001

The influence of CRNDE on LGG progression was further investigated through in vitro experiments. Three specific shRNAs targeting CRNDE named sh-CRNDE#1–3 were transfected into two LGG cell lines (U251 and SW1088), and RT-qPCR results showed that CRNDE expression was significantly reduced by sh-CRNDE (Fig. 8A, B). Next, flow cytometry was conducted to measure the apoptosis of U251 and SW1088 cells. As a result, higher apoptosis rate was found in CRNDE-knockout cells (Fig. 8C–E). This indicated that knockdown of CRNDE significantly promoted apoptosis of LGG cells. Wnt/β-catenin signaling plays a crucial role in cancer progression. Therefore, we detected the expression of β-catenin and Wnt5a in U251 and SW1088 cells. Consequently, the expression of β-catenin and Wnt5a was remarkably decreased in CRNDE-knockout LGG cells (Fig. 8F–K). Altogether, targeting CRNDE could alleviate malignant phenotypes of LGG.

## Discussion

This study determined that CRNDE was up-regulated in LGG, and the up-regulation was capable of predicting patients’ clinical outcomes and progression. CRNDE expression was positively linked to grade G3, wild-type IDH and non-codel 1p19q. LGG patients with codeletion of chromosomal arms 1p and 19q (1p/19 codeletion) usually present favorable clinical outcomes [26]. By integrating independent risk factors (CRNDE and grade), we established the nomogram that could accurately predict LGG prognosis for clinical practice. More genomic variations were found in LGG with high CRNDE expression. Moreover, up-regulated CRNDE was correlated to tumorigenic pathways, and tumor immunity (antigen processing and presentation, etc.), stromal activation, and DNA damage repair, thereby revealing the crucial roles of CRNDE in LGG.

The brain tumor microenvironment comprises many different nonneoplastic cell types, all of which exert distinct roles in the formation, maintenance, and progression of LGG [27]. Monocytes, dendritic cells, NK cells, and T cells are the dominating nonneoplastic cell types in LGG [28]. Despite the roles of T cells in targeting and eliminating tumor cells, they also exist in other states, such as tolerance, ignorance, anergy and exhaustion. Moreover, T cells act as a driver of LGG growth. Because T cells are from the blood and bone marrow sinuses, their functions as both positive and negative regulatory factors of LGG growth have ignited renewed interest in their deployment as immunotherapy drugs. A randomized trial showed that neoadjuvant vaccination with tumor-cell lysate enabled to induce effector CD8 + T cell response in LGG patients’ peripheral blood and vaccine-reactive CD8 + T cells to migrate into the tumor microenvironment [29]. Up-regulated CRNDE was linked to the enhanced infiltration of most immune cell types (especially monocytes, dendritic cells, NK cells, and T cells). Previously, LGG can be classified as six subtypes in TCGA dataset, including C1 (wound healing), C2 (IFN-γ dominant), C3 (inflammatory), C4 (lymphocyte depleted), C5 (immunologically quiet), and C6 (TGF-β dominant). Patients with high CRNDE expression displayed more C4 and C5 phenotypes. Although we assessed the response of LGG patients to immunotherapy in TCGA and CGGC cohorts, our study cannot analyze whether immunotherapy-received LGG patients with distinct CRNDE expression had distinct benefits due to the lack of expression profiles. Investigations should be undertaken in our further research to compare CRNDE with current biomarkers as well as to evaluate the association between CRNDE expression and immunotherapy in LGG patients.

CRNDE restrains chemoresistance in gastric cancer through SRSF6-mediated alternative splicing of PICALM [30]. Transfer of CRNDE in tumor-associated macrophages-derived exosomes can be attributed to cisplatin resistance in gastric cancer [31]. On the contrary, CRNDE triggers chemoresistance of colorectal cancer (CRC) through miR-181a-5p-regulated Wnt/β-catenin pathway [32]. Additionally, it promotes oxaliplatin resistance via sponging miR-136 in CRC [33]. Downregulated CRNDE attenuates drug resistance of liver cancer cells through enhancing miRNA-33a expression and reducing HMGA2 expression [34]. CRNDE facilitates ATG4B-induced autophagy as well as weakens sorafenib sensitivity in hepatocellular carcinoma (HCC) cells [35]. CRNDE promotes chemoresistance in HCC via epigenetically suppressing CUGBP Elav-like family member 2 (CELF2) and large tumor suppressor 2 (LATS2) [36]. Suppression of CRNDE attenuates proliferation and P-glycoprotein-induced multidrug resistance in acute myelocytic leukemia via Wnt/β-catenin signaling [37]. CRNDE contributes to the resistance to EGFR tyrosine kinase inhibitor in EGFR-mutant lung cancer through eIF4A3/MUC1/EGFR signaling [38]. Suppression of CRNDE heightens the sensitivity of temozolomide via modulating autophagy in glioblastoma [6]. Our study demonstrated that up-regulated CRNDE was linked to the increased sensitivity to Cisplatin, Erlotinib, Methotrexate, Paclitaxel, Camptothecin, Etoposide, Rapamycin, and Doxorubicin in LGG. Additionally, CRNDE was identified to be associated with the sensitivity to three compounds (Etoposide, Valrubicin, and Daunorubicin). This indicated that CRNDE expression was notably in relation to drug sensitivity in LGG.

This study still has several limitations. Firstly, the utilization of the two largest LGG databases inevitably results in the neglect of intratumoral heterogeneity in distinct databases. Secondly, identification of the optimal cutoff value of CRNDE expression might offer more favorable results compared with the median value of its expression. Thirdly, although we determined the associations between CRNDE expression and tumor immunity, the underlying mechanisms were still unclear.

## Conclusion

Collectively, we conducted systematic analyses of CRNDE in LGG biology. CRNDE acted as a prognostic factor of LGG. High CRNDE expression was linked to more genomic variations, tumor immunity, and therapeutic sensitivity. Hence, quantification of CRNDE expression might represent a promising approach for predicting the therapeutic benefits of LGG patients.

## Supplementary Information

**Additional file 1. **The schematic diagram of our study design.

**Additional file 2. **Validation of clinical features and prognostic value of CRNDE in CGGC and GSE16011 cohorts. (A) Differences in CRNDE expression in different clinical variables, age≤40 vs. >40; male vs. female; grade G2 vs. G3; mutant vs. wild type IDH; codel vs. non-codel 1p19q; methylated vs. unmethylated MGMT in CGGC cohort. (B) Kaplan-Meier curves of OS for LGG cases with high or low CRNDE expression in CGGC cohort. (C) ROC curves at 1-, 3- and 5-year OS for CRNDE expression in CGGC cohort. (D, E) Validation of (D) Kaplan-Meier curves and (E) ROC curves for CRNDE in GSE16011 cohort.

**Additional file 3. **Verification of the CRNDE-based nomogram for LGG in GSE16011 dataset. (A, B) Forest diagrams of uni- and multivariate analyses of CRNDE and clinical variables with patient prognosis. (C) The nomogram establishment. (D) Calibration curves for predicting 1-, 3- and 5-year survival probability. (E-G) DCA curves at (E) 1-, (F) 3- and (G) 5-year survival threshold probabilities for intuitively evaluating the nomogram’s clinical benefits and application

**Additional file 4. **Validation of the CRNDE-based nomogram for LGG in CGGC dataset. (A, B) Forest diagrams of uni- and multivariate analyses on CRNDE and clinical variables with patient survival. (C) Calibration curves of the CRNDE-based nomogram at 1-, 3- and 5-year survival. (D) ROC curves at 1-, 3- and 5-year survival. (E, F) DCA curves at (E) 3- and (F) 5-year survival threshold probabilities.

## Data Availability

The data used to support the findings of this study are included within the supplementary information files.

## Abbreviations

LGG: Low grade glioma
CRNDE: Colorectal tumor differentially expressed
lncRNA: Long noncoding RNA
FPKM: Fragments per kilobase per million
TCGA: The Cancer Genome Atlas
TPM: Transcripts per kilobase million
CNVs: Copy number variations
TMB: Tumor mutational burden
CTA: Cancer-testis antigen
mRNAsi: MRNA expression based-index
CGGC: Chinese Glioma Genome Atlas
MSigDB: Molecular Signatures Database
GSEA: Gene set enrichment analysis
HLA: Human leukocyte antigen
ESTIMATE: Estimation of STromal and Immune cells in MAlignant Tumor tissues using Expression data
TIDE: Tumor Immune Dysfunction and Exclusion
IC50: 50% Inhibiting concentration
HR: Hazard ratio
CI: Confidence interval
C-index: Concordance index
ROC: Receiver operating characteristic
OS: Overall survival
DFS: Disease-free survival
PFS: Progression-free survival
DSS: Disease-specific survival
AUC: Area under the curve
shRNA: Short hairpin RNA
