# Supplementary figures and images for: Implications of CRNDE in prognosis, tumor immunity, and therapeutic sensitivity in low grade glioma patients
Source: Cancer Cell Int. 2023 May 16;23:93. doi: 10.1186/s12935-023-02930-w (PMC10186690; doi:10.1186/s12935-023-02930-w)

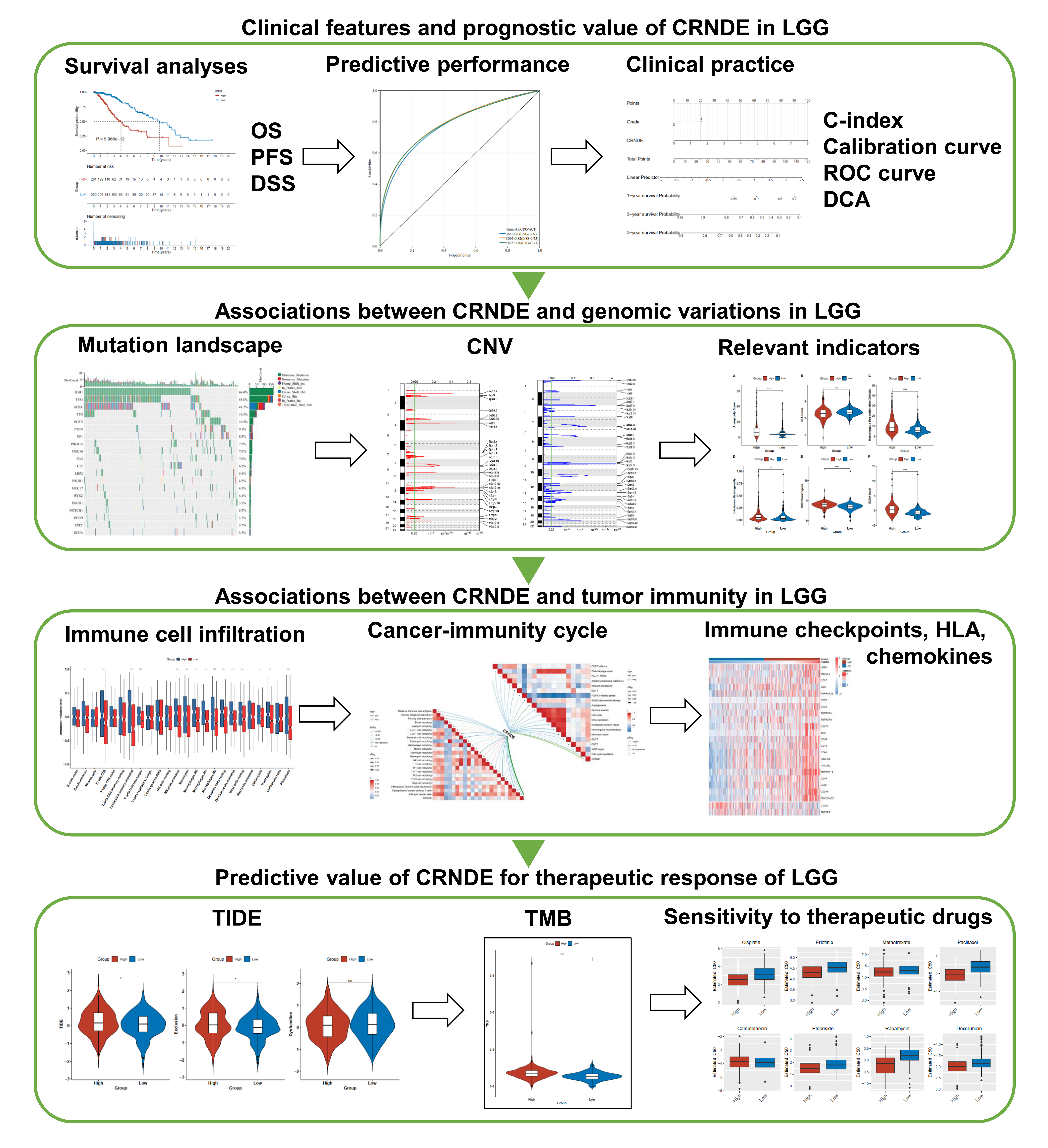

Supplement: Supplementary file 1 — Additional file 1. The schematic diagram of our study design. [file 12935_2023_2930_MOESM1_ESM.tif]

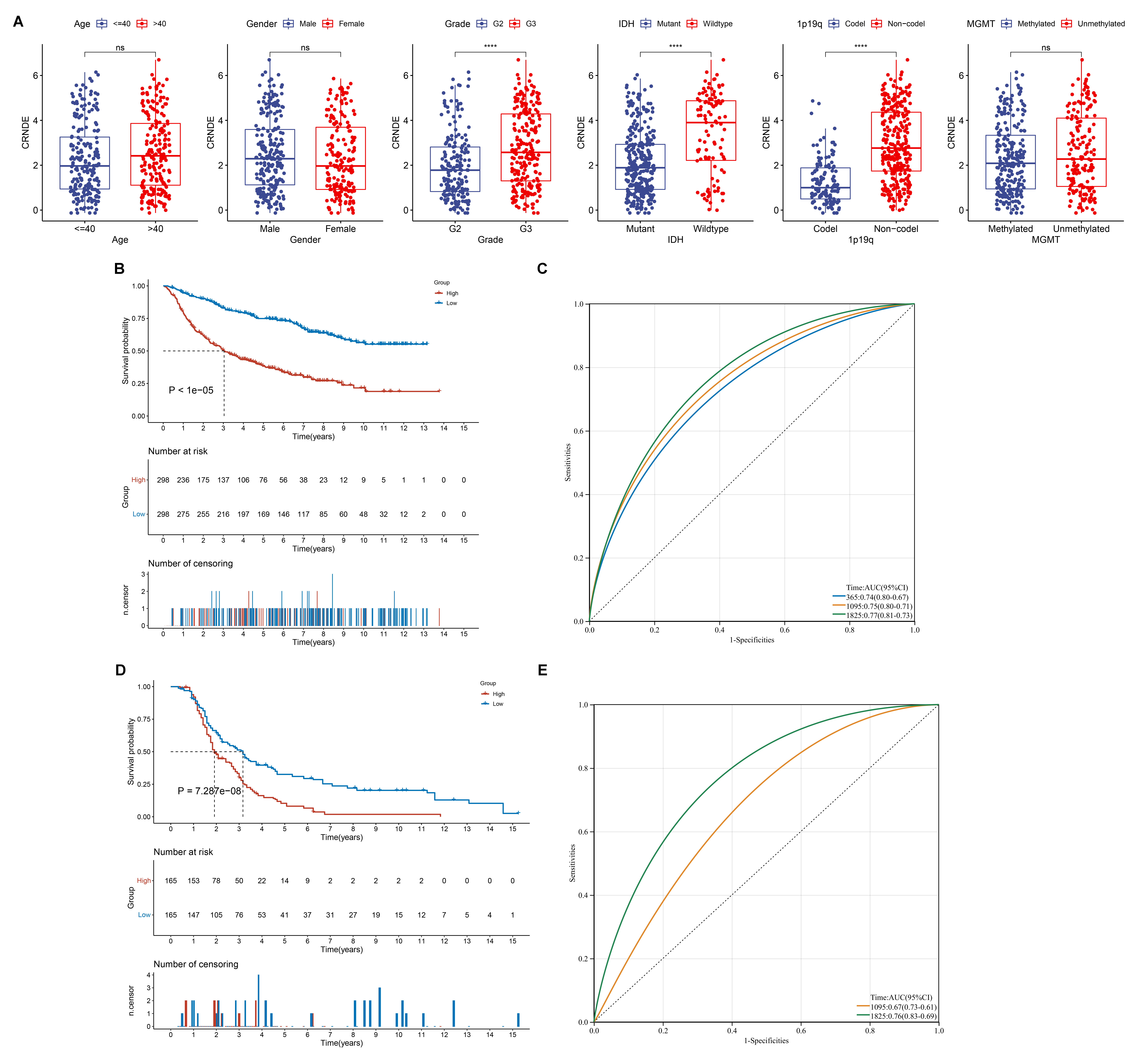

Supplement: Supplementary file 2 — Additional file 2. Validation of clinical features and prognostic value of CRNDE in CGGC and GSE16011 cohorts. (A) Differences in CRNDE expression in different clinical variables, age≤40 vs. >40; male vs. female; grade G2 vs. G3; mutant vs. wild type IDH; codel vs. non-codel 1p19q; methylated vs. unmethylated MGMT in CGGC cohort. (B) Kaplan-Meier curves of OS for LGG cases with high or low CRNDE expression in CGGC cohort. (C) ROC curves at 1-, 3- and 5-year OS for CRNDE expression in CGGC cohort. (D, E) Validation of (D) Kaplan-Meier curves and (E) ROC curves for CRNDE in GSE16011 cohort. [file 12935_2023_2930_MOESM2_ESM.tif]

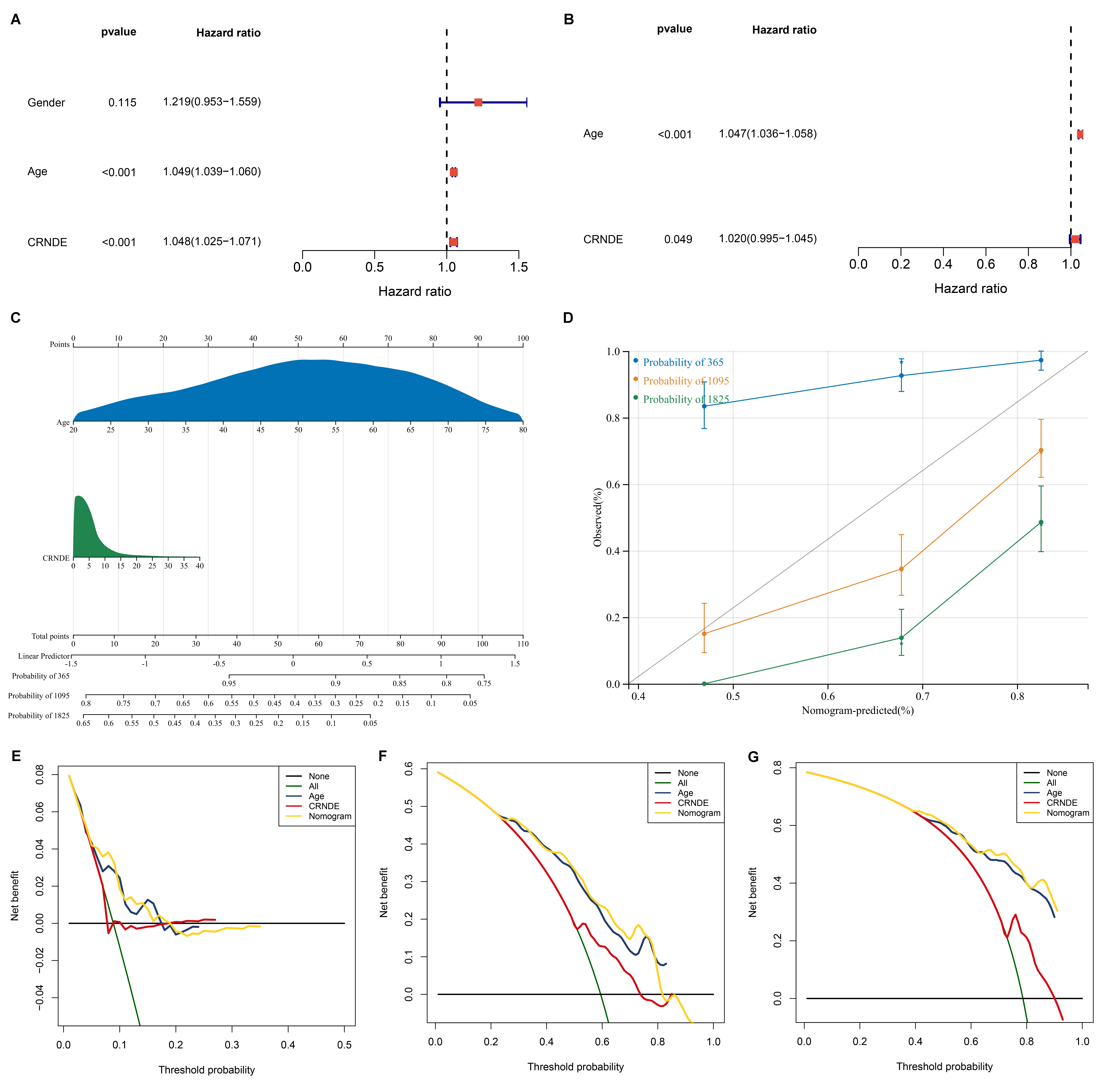

Supplement: Supplementary file 3 — Additional file 3. Verification of the CRNDE-based nomogram for LGG in GSE16011 dataset. (A, B) Forest diagrams of uni- and multivariate analyses of CRNDE and clinical variables with patient prognosis. (C) The nomogram establishment. (D) Calibration curves for predicting 1-, 3- and 5-year survival probability. (E-G) DCA curves at (E) 1-, (F) 3- and (G) 5-year survival threshold probabilities for intuitively evaluating the nomogram’s clinical benefits and application [file 12935_2023_2930_MOESM3_ESM.tif]

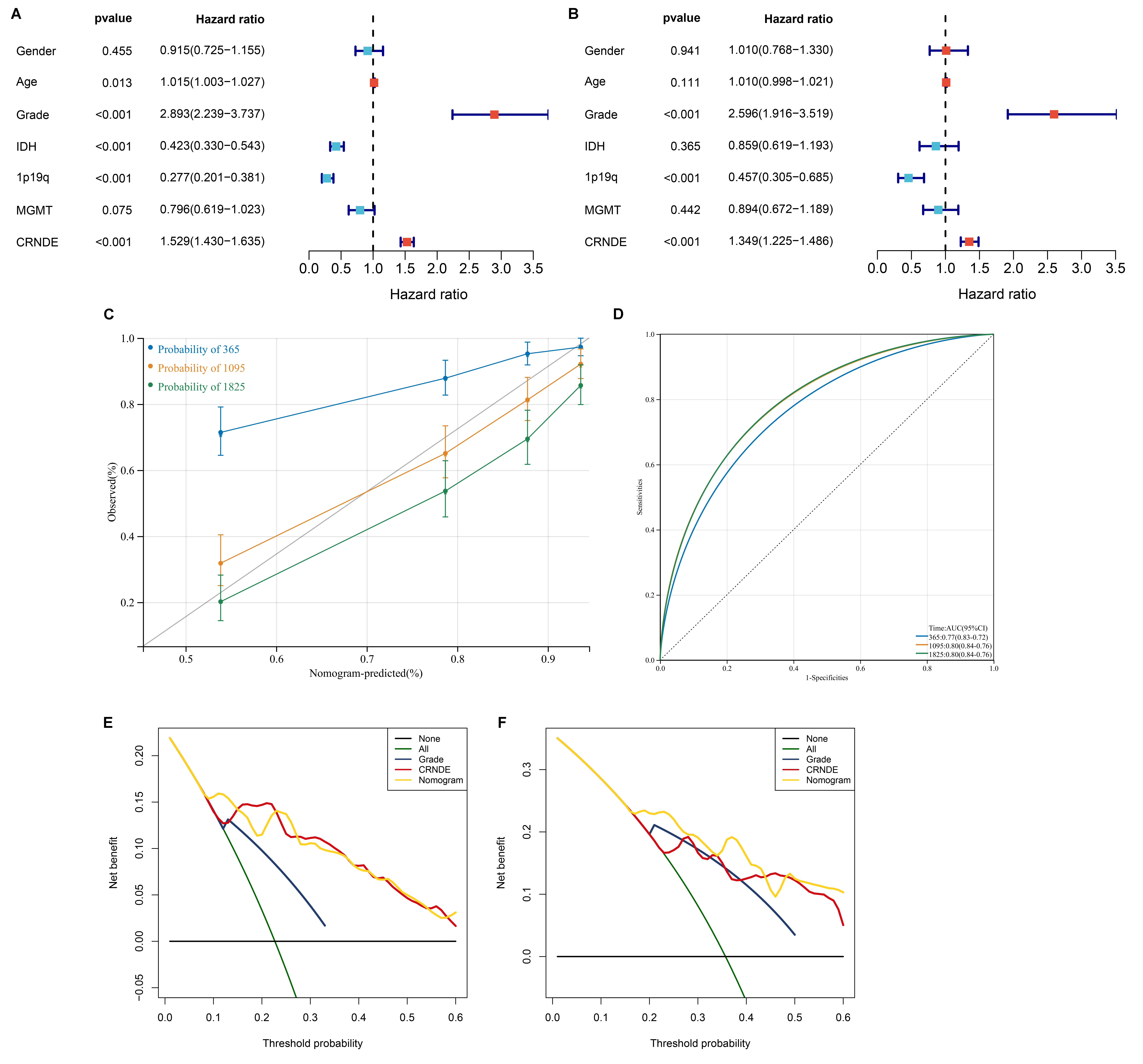

Supplement: Supplementary file 4 — Additional file 4. Validation of the CRNDE-based nomogram for LGG in CGGC dataset. (A, B) Forest diagrams of uni- and multivariate analyses on CRNDE and clinical variables with patient survival. (C) Calibration curves of the CRNDE-based nomogram at 1-, 3- and 5-year survival. (D) ROC curves at 1-, 3- and 5-year survival. (E, F) DCA curves at (E) 3- and (F) 5-year survival threshold probabilities. [file 12935_2023_2930_MOESM4_ESM.tif]
